# The Cost of Lost Productivity Due to Premature Chagas Disease-Related Mortality: Lessons from Colombia (2010–2017)

**DOI:** 10.3390/tropicalmed6010017

**Published:** 2021-01-27

**Authors:** Mario J. Olivera, Francisco Palencia-Sánchez, Martha Riaño-Casallas

**Affiliations:** 1 Grupo de Parasitología, Instituto Nacional de Salud, Bogotá 111321, D.C., Colombia; 2 Programme in Health Economics, Pontificia Universidad Javeriana, Bogotá 110231, D.C., Colombia; 3 Facultad de Medicina, Departamento de Medicina Preventiva y Social, Pontificia Universidad Javeriana, Bogotá 110231, D.C., Colombia; fpalencia@javeriana.edu.co; 4 Facultad de Ciencias Económicas, Universidad Nacional de Colombia, Bogotá 111321, D.C., Colombia; mirianoc@unal.edu.co

**Keywords:** Chagas disease, cost of illness, premature, efficiency, organizational, life expectancy

## Abstract

Background: Economic burden due to premature mortality has a negative impact not only in health systems but also in wider society. The aim of this study was to estimate the potential years of work lost (PYWL) and the productivity costs of premature mortality due to Chagas disease in Colombia from 2010 to 2017. Methods: National data on mortality (underlying cause of death) were obtained from the National Administrative Department of Statistics in Colombia between 2010 and 2017, in which Chagas disease was mentioned on the death certificate as an underlying or associated cause of death. Chagas disease as a cause of death corresponded to category B57 (Chagas disease) including all subcategories (B57.0 to B57.5), according to the Tenth Revision of the International Statistical Classification of Diseases and Related Health Problems (ICD-10). The electronic database contains the number of deaths from all causes by sex and 5-year age group. Economic data, including wages, unemployment rates, labor force participation rates and gross domestic product, were derived from the Bank of the Republic of Colombia. The human capital approach was applied to estimate both the PYWL and present value of lifetime income lost due to premature deaths. A discount rate of 3% was applied and results are presented in 2017 US dollars (USD). Results: There were 1261 deaths in the study, of which, 60% occurred in males. Premature deaths from Chagas resulted in 48,621 PYWL and a cost of USD 29 million in the present value of lifetime income forgone. Conclusion: The productivity costs of premature mortality due to Chagas disease are significant. These results provide an economic measure of the Chagas burden which can help policy makers allocate resources to continue with early detection programs.

## 1. Introduction

Chagas disease remains a serious public health problem worldwide, having serious economic and social repercussions [1]. The infection is endemic in South America and emergent in Europe and the United States [2]. This parasitic disease affects 6–7 million people worldwide, causing more than 7000 deaths each year [3]. The cost of Chagas disease was USD 13.1 million in 2017 [4].

Chagas disease generates a significant health burden for individuals and a large economic burden in low- and middle-income countries in the Americas and in some high-income countries over recent decades [4]. Among the working-age population, the economic cost of illness-related productivity losses as a result of lower productivity at work, lost workdays, and mortality can far exceed the Chagas disease-related medical costs [5].

It is important to quantify the value of the labor productivity loss due to premature mortality in measuring the economic burden of disease. Specially, this metric should be quantified for communicable diseases in that affect low- and middle-income countries. To quantify the cost of economic losses owing to premature death in the working-age population, this value is used as the indicator of years of potential productive life lost [6,7]. In this case, we focused on economic cost. Chagas disease has been associated with excess mortality [8]. The most frequently used measures of economic loss due to premature death are years of potential life lost (YPLL) and potential years of work lost (PYWL) [6,9,10,11].

Chagas disease is a clear threat not only to human health but also the level of family income and economic growth in a country, particularly in rural areas [4]. It is estimated that 752,000 working years per year are lost due to premature deaths caused by diseases in the seven countries of South America, which corresponds to USD 1208.5 million/year [5].

Despite the high prevalence of Chagas disease estimated in Colombia 2.0% (95% CI: 1.0–4.0) [12], few studies have estimated the productivity losses associated with premature deaths from this infection in the country [4]. Therefore, this study aimed to estimate the PYWL associated with premature deaths caused by Chagas disease during the period from 2010 to 2017 in Colombia.

## 2. Materials and Methods

This study was developed based on the human capital approach to estimate the costs of productivity derived from premature mortality due to Chagas disease in Colombia. Premature mortality was defined as death from Chagas disease before the age of 62 (for men) or 57 (for women), years old. The human capital approach equates the productivity lost to an individual’s wage rate and assumes that an individual produces a stream of output over a working lifetime cut short by premature death. All expenses were reported as Colombian pesos (COP) and were converted to US dollars (1 USD (US$) = 2984 COP) from 2017 [13].

### 2.1. Data Source

Numbers of deaths in 2010–2017 by 5-year age group and sex between the ages of 15 and 62 were obtained from the mortality database of the National Administrative Department of Statistics (DANE) using the Tenth Revision of the International Statistical Classification of Diseases and Related Health Problems (ICD-10) code B57, including all subcategories (B57.0 to B57.5) [14]. The database contains number of deaths of all causes by sex and 5-year age. Economic data, including wages, unemployment rates, labor force participation rates and gross domestic product (GDP), were derived from Bank of the Republic of Colombia.

### 2.2. Estimation Methods

The number of deaths that could be attributed to Chagas from 2010 to 2017 by sex was extracted, and from these, PYWL for men and women were determined across the productive age groups (between 18 and 62 years old, retire at 62 (for men) or 57 (for women)—the official pensionable age in Colombia in 2017 [15]). Premature mortality costs involved multiplying, for each death, PYWL by age- and gender-stratified gross wages from age of death until to the official pensionable age. Estimates were the adjusted probability of being in work. Wage growth was calculated at 2.5% per annum, and a discount rate of 3% annually was applied. The scenario that assessed the 2017 minimum annual salary (USD 3301 per year) was modeled. In Colombia on average the growth of the real wage was 2%. Statistical analysis was performed using Stata version 14.0 (Stata Corporation LP, College Station, TX, USA). All variables included in the study were described using the appropriate univariate statistics.

### 2.3. Sensitivity Analyses

One-way sensitivity analysis was conducted to assess the effects of varying the parameters: the wage growth rate varied from 1.5% to 3.5% to account for uncertainty over future growth in the Colombian economy, and the minimum annual salary between USD 2715 and USD 4000. In addition, the effect of extending the retirement age was explored.

## 3. Results

From 2010 to 2017, 1446 deaths of Chagas disease were recorded. Of these, 185 deaths occurred in people under 18 years of age were excluded. In total, 1261 deaths were analyzed in the study, of which 60% corresponded to males. The mean age at death was 21 years. Table 1 presents the number of deaths of all ages for males and females. PYWL was lower in women than men (18,384 vs. 30,237), overall PYWL was 48,621. It noticed that the deaths each year of the analysis period.

Table 2 demonstrates the average premature mortality per PYWL by sex from 2010 to 2017. The cost per PYWL for both sexes combined was USD 29,683,913 in the study period, and it was USD 17.3 million for males and USD 12.3 million for females from 2010 to 2017. In the case of women, they tended to have lower wages and a shorter working life.

The total cost of lost productivity due to premature mortality was 39.7% higher in males than females, although the cost per PYWL was higher in females.

Table 3 shows the cost of premature mortality sex in each year of the period.

We classified the people included according to age; people who died between the ages of 18 and 25 years were categorized as young and people above 25 years old classified as adults. Therefore, Table 4 shows the impact of the cost is bigger.

**Figure 1:**
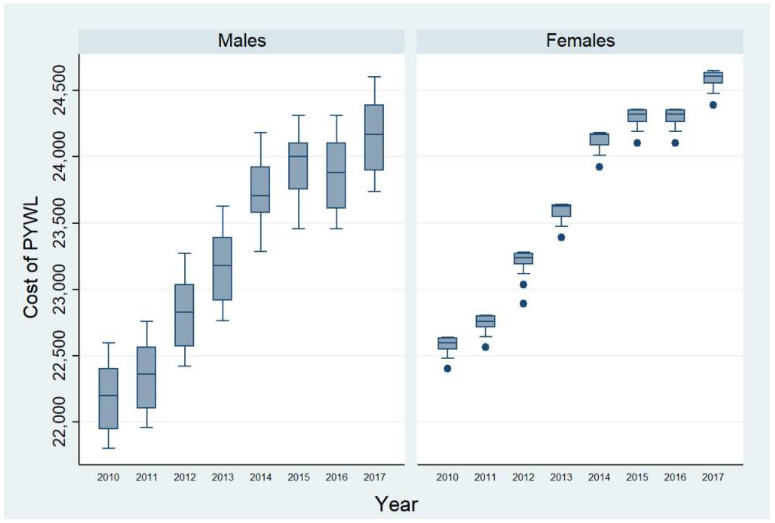
Cost of productivity lost due to premature mortality by sex (USD 2017).

Figure 1 shows a boxplot of cost of premature mortality by sex, for which the variation was higher for men per death than for women. This is because men die younger than women and they have a longer pensionable age. In the graph, the circles are outlier values of the cost of PYWL.

**Figure 2:**
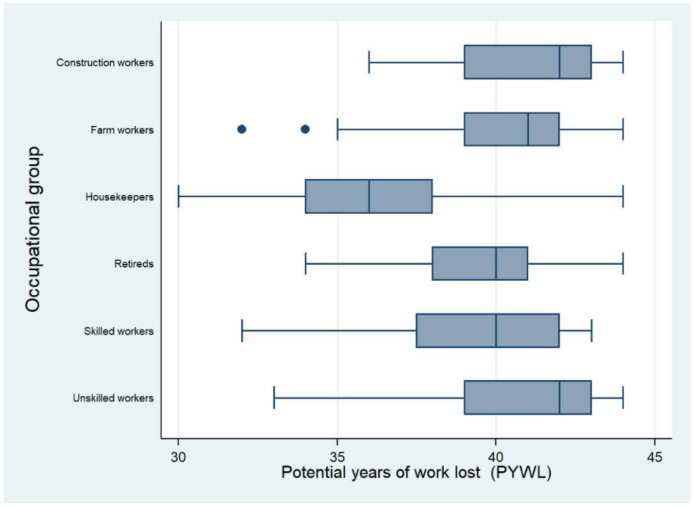
Potential Years of Work Lost (PYWL) by occupation.

Figure 2 depicts the PYWL per occupational group; construction workers, farm workers and unskilled workers were the groups with the most years lost. In the graph, the circles are outlier values of the cost of PYWL in farm workers.

## 4. Discussion

The main finding of this study was the estimation of the monetary value of the accumulated labor productivity losses during the 2010–2017 period due to deaths caused by Chagas disease in Colombia. This cost amounted to USD 29 million. Despite the magnitude of the estimated cost, the trend observed throughout the period was that of further increasing costs. However, it should be clarified that this increase in the number of deaths could be due to the strengthening of surveillance systems that allow for a better counting of deaths and to the strengthening of the health system.

In recent years, Colombia has had great social, demographic, environmental and technological transformations in a sustained manner, and despite the innumerable situations of social injustice, the living conditions of the populations have improved significantly [16,17]. However, diseases associated with contexts of social vulnerability and neglect, such as Chagas disease, still affect a considerable part of the population for example workers such as unskilled workers, farm worker and construction workers [12].

It is also worrying that the percentage of deaths from preventable Chagas disease continues to be high in the younger population. This is probably associated with barriers to timely diagnosis that persist in the country and the difficulties associated with treatment [1,18,19]. This implies support for the early detection programs [20,21].

Interestingly, 60% of the estimated losses in labor productivity can be attributed to men. This can be explained by the higher risk of death in this group and, on the other hand, by the fact that employment rates and wages were higher for men than for women. It could also be related to the difference between men and women. These results are concordantwith previous studies that have consistently reported that men have a higher risk of death than women [22,23].

Previous studies have tried to estimate the social impact of premature deaths on workers suffering from Chagas disease, but over a short time period [4]. On the other hand, some research has delved into the loss of health-related quality of life caused by the consequences of the disease [24,25]. The strengthening and implementation of public policies aimed at eliminating barriers to early diagnosis and treatment of Chagas disease can impact on the reduction of mortality [26].

It is important to note that the theoretical approach used in the present study is ttheory of human capital [27]. The main alternative approach is the so-called friction-cost method [28]. Although the methodological discussion on the strengths and weaknesses of both approaches has been intense, there is still no agreement on which is best [27]. In this study, the human capital approach was chosen due to its greater anchorage with economic theory and it is the most widely used method in the scientific literature on disease cost studies.

The main limitations include, firstly, the real wages of people killed by Chagas disease (estimated from the average wage in Colombia) were not considered. Second, there was also no information on whether the deceased worked or not (the average employment rates adjusted for age and sex). Third, the mortality database might have been vastly underreported in official statistics.

## 5. Conclusions

Reducing premature and preventable deaths from Chagas disease is a key health goal in the ten-year plan for Colombian public health. The size of the economic impact and the burden on society due to premature deaths from Chagas disease reinforces the need to continue investing in early detection programs, as well as initiatives that promote prosperity and well-being for all.

## Figures and Tables

**Table 1:** The number of deaths and estimated Potential Years of Work Lost (PYWL) by sex from 2010 to 2017.

**Year**	**2010**	**2011**	**2012**	**2013**	**2014**	**2015**	**2016**	**2017**	**Total**
**Number of Deaths**									
Males	102	87	101	110	113	93	132	128	866
Females	58	51	59	71	79	85	92	85	580
Total	160	138	160	181	192	178	224	213	1446
**Deaths at Working Age**									
Males	84	69	88	95	93	83	117	112	741
Females	52	45	51	64	72	76	84	76	520
Total	136	114	139	159	165	159	201	188	1261
**PYWL**									
Males	3441	2822	3594	3900	3777	3348	4772	4583	30,237
Females	1854	1593	1824	2263	2521	2682	2968	2679	18,384
Total	5295	4415	5418	6163	6298	6030	7740	7262	48,621

**Table 2:** Premature mortality cost by sex per death and per Potential Years of Work Lost (PYWL) (USD 2017).

	Total Premature Mortality Cost	% of the Total	Premature Mortality Cost per Death	Premature Mortality Cost per PYWL
Males	17,301,237	58	23,348	572
Females	12,382,676	42	23,813	674
Total	29,683,913	100	23,540	611

**Table 3:** Premature mortality cost per sex 2010–2017 (USD 2017).

Year	2010	2011	2012	2013	2014	2015	2016	2017
Males	1,862,873	1,541,799	2,008,175	2,198,782	2,206,153	1,986,068	2,793,321	2,704,067
Females	1,174,136	1,023,484	1,183,187	1,509,232	1,736,916	1,846,584	2,040,400	1,868,737
Total	3,037,009	2,565,283	3,191,362	3,708,014	3,943,069	3,832,652	4,833,722	4,572,803

**Table 4:** Premature mortality cost per group age 2010–2017 (USD 2017).

Age Group/Year	2010	2011	2012	2013	2014	2015	2016	2017
Adults	22,597	68,203	46,165	47,106	96,491	72,932	170,012	98,320
Young	3,014,412	2,497,081	3,145,197	3,660,908	3,846,578	3,759,719	4,663,710	4,474,483
